# GO-based Functional Dissimilarity of Gene Sets

**DOI:** 10.1186/1471-2105-12-360

**Published:** 2011-09-01

**Authors:** Norberto Díaz-Díaz, Jesús S Aguilar-Ruiz

**Affiliations:** 1 School of Engineering, Pablo de Olavide University, Seville, Spain

## Abstract

**Background:**

The Gene Ontology (GO) provides a controlled vocabulary for describing the functions of genes and can be used to evaluate the functional coherence of gene sets. Many functional coherence measures consider each pair of gene functions in a set and produce an output based on all pairwise distances. A single gene can encode multiple proteins that may differ in function. For each functionality, other proteins that exhibit the same activity may also participate. Therefore, an identification of the most common function for all of the genes involved in a biological process is important in evaluating the functional similarity of groups of genes and a quantification of functional coherence can helps to clarify the role of a group of genes working together.

**Results:**

To implement this approach to functional assessment, we present GFD (GO-based Functional Dissimilarity), a novel dissimilarity measure for evaluating groups of genes based on the most relevant functions of the whole set. The measure assigns a numerical value to the gene set for each of the three GO sub-ontologies.

**Conclusions:**

Results show that GFD performs robustly when applied to gene set of known functionality (extracted from KEGG). It performs particularly well on randomly generated gene sets. An ROC analysis reveals that the performance of GFD in evaluating the functional dissimilarity of gene sets is very satisfactory. A comparative analysis against other functional measures, such as GS^2 ^and those presented by Resnik and Wang, also demonstrates the robustness of GFD.

## Background

The Gene Ontology (GO) [1] is a cross-species, controlled vocabulary describing three major functional characteristics of gene products: molecular function, cellular component and biological process. The information is structured as a directed acyclic graph for each sub-ontology. Each node in the graph represents a class of genes identified by a GO-term. Each edge represents the relationship between the terms it connects, which can be "is a", "is a part of", or "regulates", meaning that a child class is either a part of the parent, is a more specific example of the parent class or is regulated (positively or negatively) by the parent, respectively.

The GO provides a controlled vocabulary for describing gene product functions and can be used to evaluate the functional coherence of gene sets. There are two major approaches for analyzing GO-based gene annotations: enrichment tools and semantic similarity measures.

### Enrichment tools

Enrichment tools are used to determine the common features of a set of genes by examining annotations and finding GO-terms that they share to a significant extent. For example, the To-Go java navigation tool [2] allows users to navigate the GO with various kinds of queries. There are also enrichment tools that provide ontological analyses with different statistical models, including the hypergeometric, binomial, Pearsons's chi-squared and Fisher's exact tests [3]. GeneTools [4] is a web service that provides access to several databases such as UniGene [5], Entrez Gene [6], Swiss-Prot [7] and Gene Ontology. It includes a tool for visualisation and statistical hypothesis testing to assess the similarity of GO-term annotations in different gene lists. The local graph structure of GO hierarchy is available from GOLEM (Gene Ontology Local Exploration Maps) [8]. It also supports rapid analysis of an input list of genes to find enriched GO terms. FuncAssociate [9], takes a list of genes as an input and indicates whether a significant number of the genes share a certain GO term. Based on the same concept of GO enrichment, the tools Bingo [10] and Ease (DAVID) [11] take a set of genes and identify the saturated terms.

### Semantic similarity measures

In general, GO-based enrichment tools are used to analyse GO term in large-scale gene sets. However, while they all determine whether an observed number of GO annotations in a set is significant, they lack a quantitative similarity measure that would allow for a complete comparable analysis of gene sets or models produced by microarray analysis. Although many enrichment tools are used for analyzing microarray data and give a level of significance for the designed enrichment, they only inform about the data distribution and do not give information on the inherent relationship, which is critical at comparing sets of genes.

To address this issue, semantic similarity methods have been developed. This category comprises GO-term, gene-product and gene-similarity approaches. GO-term similarity approaches have been presented by Couto et al. [12], Kriventseva et al. [13], and Lee et al. [14] and are based on measures [15-17] originally developed for other semantic taxonomies. These measures determine the similarity of two GO-terms using the information content for their lowest common ancestor. Guo et al. [18-20] evaluated these methods and showed that Resnik's method is better than the others in terms of correlating gene sequence similarities and gene expression profiles. del Pozo et al. [21] proposed a new method for quantifying functional distances between GO terms. Their method is based on the simultaneous occurrence of terms in the same set of Interpro [22] entries does not rely on the structure of the GO itself. In the same vein, Wang et al. [23] presented a method to encode a GO-term's semantics as a numeric value by aggregating the semantic contributions of ancestor terms. This proposal was used to develop a clustering tool to study the genes in pathways retrieved from the Saccharomyces Genome Database (SGD), and the clustering results showed that Wang's method is more consistent than Resnik's method.

The relationships of gene-products are also of interest to researchers. Until recently, the most common methods for measuring gene-product functional similarity were pairwise approaches based on GO-term. Lord et al. [24] were the first to apply this methodology, using the average of all pairwise similarities. The same approach was used by Liu et al. [25], Azuaje et al. [26] and Chagoyen et al. [27]. Lee et al. [14] and Guo et al. [28] used the maximum of all pairwise similarities; whereas Brameier and Wiuf [29] and Wu et al. [30] used a composite average in which only the best-matching term pairs are considered (best-match average). Tao et al. [31] proposed a variant, in which only those pairwise measures that exceed a threshold were considered. The performances of these pairwise-based measures were tested by Xu et al. [32]. They concluded that the method based on the best-match average consistently gave the best performance out of all of the tests that were studied.

Other methods exist for measuring gene-product functional similarity that are not based on pairwise approaches. Lerman and Shakhnovich [33] presented several manifold-embedding techniques for computing distances between GO functional annotations and for estimating functional distances between protein domains. Likewise, Schlicker et al. [34,35] proposed a method for measuring functional similarity by combining different ontologies to produce a single similarity score. Their method, named *FunSim*, is based on Schlicker's measure (*sim_Rel_*), which combines both of the Lin and Resnik GO-term similarity measures.

Bastos et al. [36] proposed three different measures: *GO_ocurrence _*to measure the functional coherence of a list of gene products; *GO_score _*to indicate how well a cluster of genes has been functionally characterised; and *GO_center _*to provide a measure of how many of the cluster's functional annotations are captured by the center of the cluster. Zheng and Lu [37] developed a measure to determine the overall functional coherence of a group of proteins by using the semantic similarity of the biomedical literature associated with the proteins.

The methods presented above measure the similarity of a pair of GO terms, a measurement that can be extended to a set of gene products or a pair of genes. The approaches that address the functional coherence of a gene set do not simply select the most common function found within the set. Instead, the gene set coherence is determined as a function of the similarity of all the pairs of genes within the set [23]. Recently, Ruths et al. [38] proposed a GO-based measure of functional similarity for gene sets, named GS^2^. This measure quantifies the similarity of a set of genes by averaging their individual contributions. Each gene is compared to the other genes in the set by calculating how closely the gene's annotation match the annotations of the others. GS^2 ^was compared to the GO pairwise measure of Wang et al. [23] by extending Wang's measure to average the contributions of all gene pairs. The comparison showed that GS^2 ^generates results more quickly and with comparable quality. To our knowledge, GS^2 ^was the first method used to determine the functional similarity of a gene set by using an entire set of genes. Later, Richards et al. [39] proposed another GO-based measure to evaluate the functional coherence of gene sets. These measures are based on the topological properties of graphs comprised of genes and their GO annotations, and they consider the enrichment of annotations and the relationships among annotations when determining the significance of functional coherence. Unlike our approach, the method of Richards et al. considers all of the annotations with equal weight. The reader is refered to reference [40] for a survey of semantic similarity measures.

Although these similarity measures have been used for different applications [40], such as comparing gene products with different functions or predicting gene product functions, they face a major limitation when confronted with genes that are involved in several functions. For such genes, the current tools give equal weight to all of the biological functions and it is not possible to single out the most relevant ones by considering the context of the other genes involved [3].

In this work, we propose a novel method for measuring gene set dissimilarity by weighting the most cohesive (common and specific) functions based on the global behaviour of the whole set of input genes. This measure, named GFD, is based on the Gene Ontology, and it assigns a numerical value to a gene set for each of the three GO ontologies.

## Method

GFD is based on an adaptation of the GO-tree structure presented in [14]. The structure is used to develop a novel GO-term dissimilarity measure for use in calculating gene set dissimilarity. To our knowledge, this is the first report of a measure that evaluates a gene set by taking into account the most cohesive function found in the set. The method involves searching for the most specific function for each gene that is also similar to the other functions found in the gene set.

**Figure 1:**
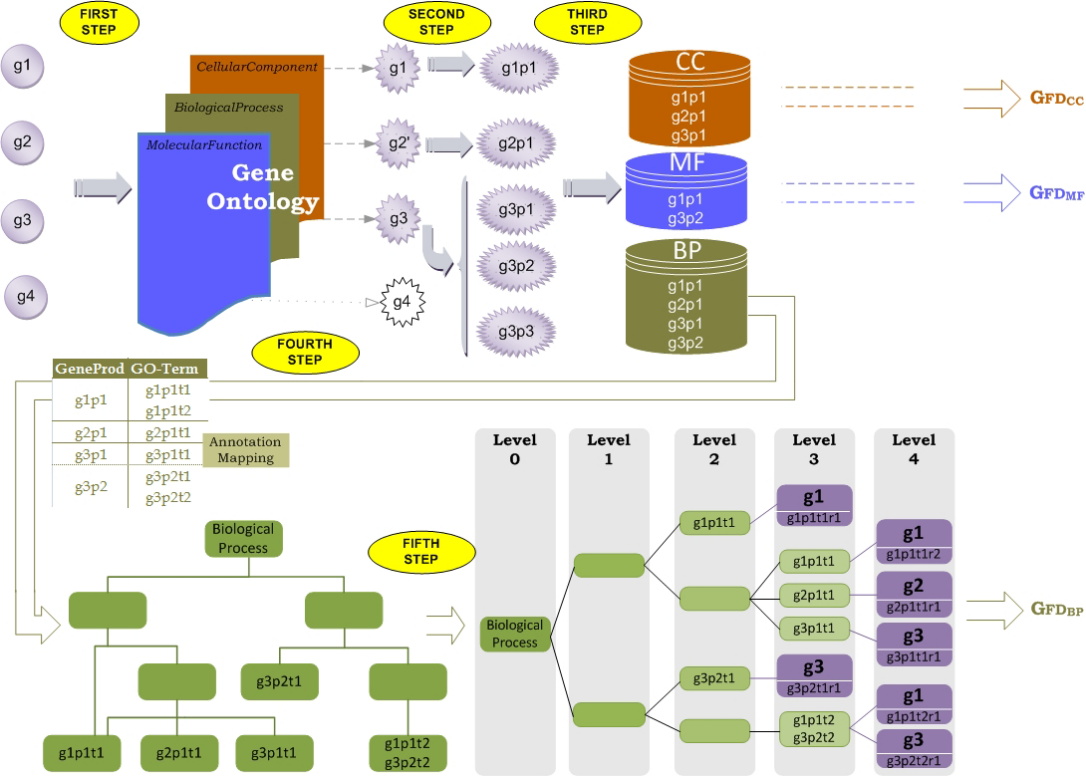
**Overall scheme of the method**. Overall scheme of the method used to calculate the gene-set functional dissimilarity measure GFD. The first three steps are used for all three ontologies. The last two steps are only illustrated for the Biological Process ontology.

The methodology is outlined in Figure 1, which presents an example of a set of four genes. The calculation of GFD entails five consecutive steps, which are described below.

### First step: Gene Identification

The first step consists of finding the representative of each input gene in the GO. Let us assume that the GO maps to Θ genes for a specific organism. Let *A *be the set of genes to be evaluated. Each *g *∈ *A *is searched for in Θ, and if the search is unsuccessful, *g *is transformed into a synonym *g' *by using the gene synonym information given in the GO annotation [41]. That is, the initial set of genes *A *= {*g*_1_,..., *g_n_*} is transformed into $A^{\prime }=\left\{g_{1}^{\prime },\ldots ,g_{n^{\prime }}^{\prime }\right\}$, where each *g' *is present in Θ. However, the gene is removed if no synonym exists. For instance, in Figure 1, genes *g*1 and *g*3 were found in the GO, gene *g*2 was transformed into a synonym (*g*2'), and gene *g*4 was not found.

### Second step: Gene-function Identification

The second step consists of identifying the function of the genes in a set. Each gene is transformed into the different proteins encoded by the gene (*the gene products*) according to the Entrez Gene database [6]. Thus, a set of gene products *H*(*i*) = {*g_i_p*_1_,..., *g_i_p_m_*} is associated with each $g_{i}^{\prime }\in A^{\prime }$. Continuing with the previous example, *g*_1 _encodes the *g*_1_*p*_1 _protein, *g*_2 _encodes the *g*_2_*p*_1 _protein, and *g*_3 _encodes *g*_3_*p*_1_, *g*_3_*p*_2 _and *g*_3_*p*_3_.

### Third step: Gene-product Filtering

In this step, each gene function is filtered by the three GO domains. The proteins chosen in the previous step are removed from those domains in which they are not involved, and are otherwise selected. Thus, in Figure 1, the *g*_1_*p*_1 _protein, encoded by gene *g*_1_, takes part in all three ontologies; the protein encoded by gene *g*_2 _(*g*_2_*p*_1_) is presented in the *Biological Process *and *Cellular Component *ontologies; and gene *g*_3 _is represented by protein *g*_3_*p*_1 _in the *Molecular Function *ontology, by the *g*_3_*p*_1 _and *g*_3_*p*_2 _proteins in the *Biological Process*, and by the *g*_3_*p*_1 _protein in the *Cellular Component *ontology. Once the input genes have been transformed into their biological functionalities and these have been filtered through each domain, the next steps must be repeated for the three ontologies, vielding three different results (one per ontology). For the sake of clarity, we will only consider the *Biological Process *ontology in the following descriptions and examples.

### Fourth step: Gene-product Annotation Search

For each ontology, the annotations of each protein are examined. A single protein can be associated with or located in one or more cellular components, it is active in one or more biological processes, during which it might perform several molecular functions. This feature is accounted for in the GO: each functional annotation is identified by a unique *GO term*.

In Figure 1, the annotations of each protein in the *Biological Process *ontology are depicted. For each *g_i_p_j_*, a set of GO terms is obtained, i.e., *H*(*i*, *j*) = {*g_i_p_j_t*_1_,..., *g_i_p_j_t_q_*}. For example, protein *g*_1_*p*_1 _has two different terms in the *Biological Process *domain. Both functionalities are used for the *g*_1_*p*_1_*t*_1 _and *g*_1_*p*_1_*t*_2 _GO terms.

### Fifth step: Gene-product Functionality

At this stage, each functional annotation in the GO has been indentified. The GO directed acyclic graph (DAG) is used, but only the "is a" relationships are considered. Our approach does not use the "part of" relationships for three reasons: a) we would like to compare results among the three domains, and the *Molecular Function *ontology does not have the "part of" relationship; b) the "part of" relationship is used in the biological process ontology when the child node is an instance of only a portion of the parent process; c) the three ontologies are now "is a" complete, meaning that every term has a path to the root node that passes solely through "is a" relationships. In Figure 1, the *g*_1_*p*_1_*t*_2 _GO term is identical to the *g*_3_*p*_2_*t*_2 _term. Therefore, both functions are located in the same node of the GO DAG. Next, the retrieved information is transformed into a tree structure (*GO tree*). A node will be present in the GO tree for each path that exists in the DAG from that node to the root. For instance, in Figure 1, *g*_1_*p*_1_*t*_1 _has two ways to reach the *Biological Process *root node, so this node is duplicated in the resulting GO tree.

Once the tree structure is built, the input genes are added to the GO tree as leaf nodes. These node positions designate the functional annotations found among the gene set. Each leaf node position is determined according to both the GO term and the protein product of the gene. A gene can be present in different leaves, which are different *representations *of the gene from different domains. Each GO-term *g_i_p_j_t_k _*will have a number of representations in the GO (the path from the GO term to the root), as it can be present in different places within the GO tree. This set of representations is denoted by *H*(*i*, *j*, *k*) = {*g_i_p_j_t_k_r*_1_,..., *g_i_p_j_t_k_r_s_*}, where *r*_1 _... *r_s _*denote the representations of term *g_i_p_j_t_k_*.

After the GO tree is constructed, the input genes can be evaluated. At this point, the initial information *A *= {*g*_1_,..., *g*_4_} has been transformed into three representations of *g*_1_, one representation of *g*_2_, and three representations of *g*_3_, each of which is located in a structure that also provides information itself. The gene set functional measure GFD, which is described in detail below, is based on the gene-representation similarity and is supported by the GO-tree structure.

### Gene-Representation Functional Dissimilarity

Let *r_α _*and *r_β _*be two gene representations. The dissimilarity between them is given by:

(1) $$R\left(r_{\alpha },r_{\beta }\right)=\frac{length\left(r_{\alpha },r_{\beta }\right)}{depth\left(r_{\alpha }\right)+depth\left(r_{\beta }\right)}$$

where *length*(*r_α_*, *r_β_*) denotes the minimum number of nodes separating *r_α _*from *r_β _*in the GO tree (i.e., the number of nodes in the path from *r_α _*to *r_β_*) and *depth *indicates the level of representation in the GO tree. From a biological point of view, *length *indicates the functional relationship of the two GO terms, whereas *depth *indicates the level of specificity of the representation. Thus, the measure penalises gene-representation pairs that are widely separated, and it rewards specialisation. This measure provides values between 0 and 1, where values close to 0 mean "similar", and values near 1 mean "dissimilar".

Two gene representations, *r_α _*and *r_β_*, present the best similarity when they share the same parent (*length*(*r_α_*, *r_β_*) = 1) and their depths are the maximum (*depth*(*r_α_*) = *depth*(*r_β_*) = *k*). In this case, their functional dissimilarity is:

$$R\left(r_{\alpha },r_{\beta }\right)=\frac{1}{k+k}\approx 0$$

In contrast, the worst case occurs when two gene representations are low in the GO tree (*depth*(*r_α_*) = *depth*(*r_β_*) = *k*), and they do not share any ancestor node other than the root node (*length*(*r_α_*, *r_β_*) = *k *+ *k *- 1):

$$R\left(r_{\alpha },r_{\beta }\right)=\frac{2k-1}{2k}\approx 1$$

For example,

$$\begin{array}{c} R\left(g_{1}p_{1}t_{2}r_{1},g_{3}p_{2}t_{2}r_{1}\right)=\frac{1}{4+4}=0.125\\ R\left(g_{1}p_{1}t_{1}r_{2},g_{3}p_{2}t_{2}r_{1}\right)=\frac{7}{4+4}=0.875 \end{array}$$

For this set of genes, the minimum and maximum values for *R *are 0.125 and 0.875, respectively.

### Functional Dissimilarity Measure

The functional dissimilarity is based on the gene-representation dissimilarity described above. Our approach extrapolates the gene-representation dissimilarity measure to evaluate gene set homogeneity. Let *A *be a set of genes {*g*_1_, *g*_2_,..., *g_n_*}. The representation set for a gene *g_i _*is given by *T*(*g_i_*), as shown in Equation 2, (see *H*(*i*, *j*, *k*) in the fifth step).

(2) $$T\left(g_{i}\right)=\underset{\begin{array}{c} j\in H\left(i\right)\\ k\in H\left(i,j\right) \end{array}}{⋃}H\left(i,j,k\right)$$

The Cartesian product *P*(*A*) = *T*(*g*_1_) × *T*(*g*_2_) × ... × *T*(*g_n_*) defines the set of all possible sets of representations. The dissimilarity *S *of a representation set *p *∈ *P *is given by Equation 3, where *R *is the dissimilarity of two gene representations as calculated by Equation 1. Note that |*p*| = |*A*|.

(3) $$S\left(p\right)=\frac{1}{\left(\begin{array}{c} \begin{array}{c} \left|p\right|\\ 2 \end{array} \end{array}\right)}\underset{\forall \delta ,\gamma |0<\delta <\gamma \leq |p|}{\sum }R\left(p\left[\delta \right],p\left[\gamma \right]\right)$$

Finally, the GO-based functional dissimilarity, GFD, is the minimum dissimilarity for all possible representation sets for a given set of genes *A*.

(4) $$GFD\left(A\right)=\underset{p\in P\left(A\right)}{\min}S\left(p\right)$$

In Figure 1 there are seven representations (three for *T*(*g*_1_), one for *T*(*g*_2_), and three for *T *(*g*_3_)), which can generate nine possible sets of representations (3 × 1 × 3), so |*P*(*A*)| = 9. There are two optimal representations for *g*_1 _and another two for *g*_3_, which yield four possible optimal configurations. However, there is only one optimal functional combination according to the cohesive function of all genes. By randomly selecting, we could obtain the worst case (*S*(*g*_1_*p*_1_*t*_1_*r*_1_, *g*_2_*p*_1_*t*_1_*r*_1_, *g*_3_*p*_2_*t*_2_*r*_1_) = 0.768), in contrast to the best case (*S*(*g*_1_*p*_1_*t*_1_*r*_2_, *g*_2_*p*_1_*t*_1_*r*_1_, *g*_3_*p*_1_*t*_1_*r*_1_) = 0.428).

It is worth noting that our approach does not select the best GO term for each gene individually; instead, it searches for the most common and specific function for the whole set of genes. In this sense, GFD is quite different from GS^2^, because it only selects one function per gene (the most globally cohesive function), whereas GS^2 ^considers all of the gene functions.

## Results and Discussion

### ROC analysis

The performance of our approach was tested by comparing it to three different measures: an information content-based measure (Resnik [17]); a hybrid (node- and edge-based) measure (Wang [23]); and GS^2 ^[38], the first measure reported to efficiently evaluate sets of genes instead of pairs of genes or GO terms. Both Resnik and Wang's measures for terms were calculated using their implementations in Bioconductor [42] and extrapolated to gene sets using the best-match average approach. The GS^2 ^source was downloaded from the website referenced in [38].

ROC analyses have been widely used in the literature [32] because they can be used to score the performance of classifiers and rankers as a trade-off between sensitivity or as a true positive rate and false positive rate. In addition, the area under the ROC curve is presented, as it provides information about the level of randomness of the approach.

Two data sets were used: sets with and without functional coherence. Both data sets were generated according to the information stored in KEGG. KEGG[43] is a database of biological systems that integrates genomic, chemical and systemic function information. This database offers genomic information about several hundred organisms, from which we selected *Saccharomyces cerevisiae *(SCE). All of the metabolic pathways from SCE were used as examples of gene sets with functional coherence. A cluster of genes was associated with each pathway. The data set without functional coherence was designed to be the same size as the functional coherence, but the genes within the clusters were randomly generated. Thus, for each pathway, we have two gene clusters of size *k*: one with genes involved in the same pathway and another with randomly generated genes.

**Figure 2:**
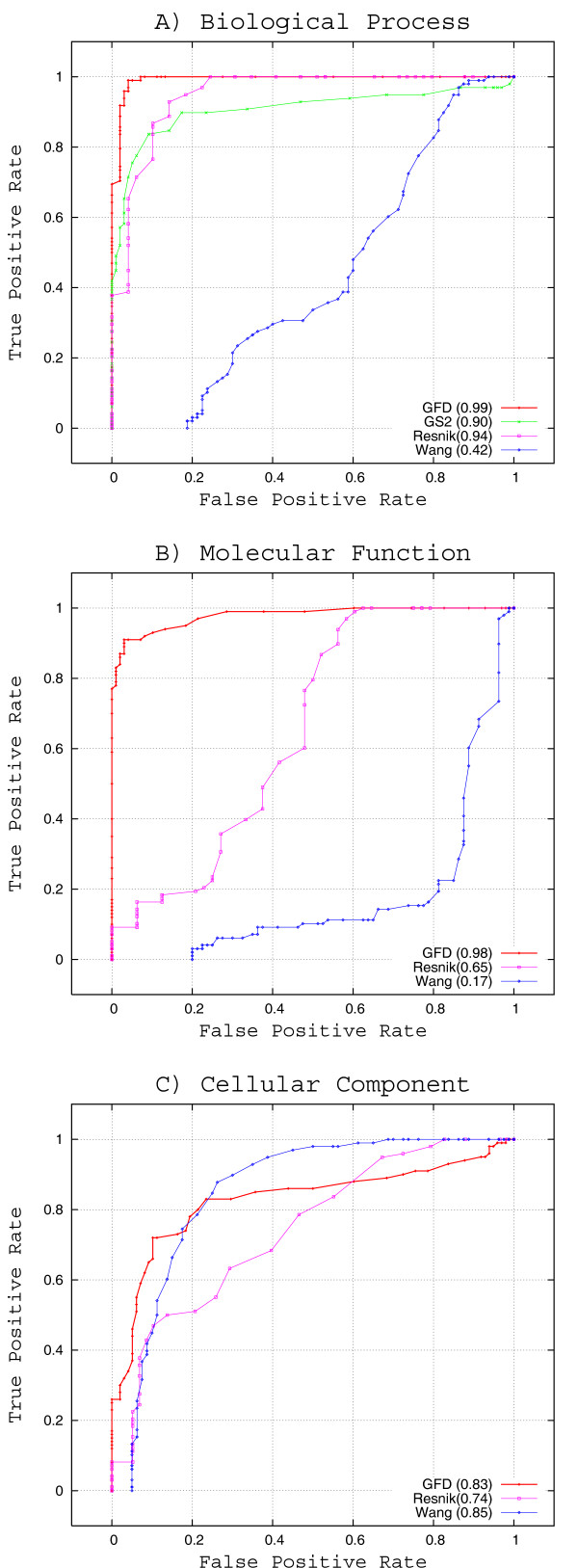
**ROC Analysis**. ROC analysis for the GFD, GS^2^, Wang and Resnik approaches, as applied to each of three GO ontologies.The area under the ROC curve is indicated in brackets.

ROC analysis was performed for the three GO ontologies. In particular, the GFD, Resnik and Wang methods were compared for the three ontologies, whereas GS^2 ^was only used for the *Biological Process *ontology because this measure can-not provide values for the other two ontologies. The ROC curve was plotted over the interval [0, 1] with increments of 0.01, as illustrated in Figure 2. The area under the ROC curve (AUC) is enclosed in brackets.

GFD shows similar and satisfactory behaviour for the three ontologies. The Resnik and Wang methods perform differently. For the *Biological Process *ontology, only Wang's approach performs worse than expected, due to its false positive rate. The AUC is above 0.90 for most measures, except for Wang's, which seems to be random (below 0.5). For the *Molecular Function *ontology, our approach is excellent, with an AUC of about 0.98, which is much greater than that of Resnik (0.65) or Wang's approach(0.17). For the *Cellular Component *ontology, the performances of the three measures are similar.

Although a biological process is not equivalent to a pathway, these concepts are very similar. For example, *Cell cycle *pathway (sce:04111) is directly related to "mitotic cell cycle" GO-term according to the information stored in KEGG. Thus, genes within the same pathway must be similar in the Biological Process ontology. However, those genes do not have to be similar in all cases under the Cellular Component ontology since they can be located in different places of cell for some pathways. For example, Cell cycle genes related to transcription are located in the nucleus while those related to translation are in the ribosome. Hence, the results obtained in this ontology are not sufficiently consistent in order to compare the performance of the different approaches. Finally, genes in the same pathway also have to be similar in the Molecular Function ontology. This ontology describes types of activities, some of which are present in the pathway describing the process. This is crucial in our study since our approach select the most cohesive function among the genes. In contrast, Wang and Resnik approaches are based on the best-match average where the same functionality may not neccessarily be selected to compute the overall similarity of the set of genes. This will cause a high false positive rate (FPR). GFD only uses one GO-term to evaluate the similarity of a gene in relation to the rest, while the other approaches can select different GO-terms to measure the similarity of a gene with regard to the other genes. This is the main reason for the poor performance of the Wang and Resnik in Molecular Function compaired to Biological Process. In general, the ROC analysis shows the robustness of GFD and demonstrates the effectiveness of the approach in evaluating the most cohesive functional annotation of a set of genes.

### Computational analysis

The gene set functional coherence measure proposed here is based on calculating the dissimilarity of all possible input gene-representation combinations. If the input set has *n *genes and each gene encodes *p *gene products, then each gene product supports *t *GO terms in each ontology, and the average number of gene representations per GO term is *r*. The computational order of the similarity measure is:

$$T(n)\in \Theta ({\left(p\times t\times r\right)}^{n}\times n^{2})=\Theta (K^{n}\times n^{2})$$

where *K *= *p *× *t *× *r *is the number of gene representations per gene. *K^n ^*is the number of different gene representation sets for each input gene. The number- of gene-representation pairs is *n*^2^. Consequently, the exhaustive calculation of GFD has a high computational complexity, making it *intractable *for large data sets. As the homogeneity measure for any set of genes should be calculated in an efficient way, we introduce a heuristic technique based on the Voronoi Diagram concept [44] that reduces the complexity from exponential to polynomial order. For each node in the GO tree, the nearest representation of each gene for that node is obtained (according to R in Eq. 1). Thus *T*(*g_i_*) (see Eq. 2) represents the set with a unique representation for each node (the nearest from gene i to the node). Once the nearest representation of each gene is found for each node, the dissimilarity values of the nodes are calculated (Eq. 3). Finally, the smallest value found for *S *is used as the GFD value.

To explore the effect of heuristics on the computational cost, we analysed the well-known cell cycle pathway (*sce:04111*) from *Saccharomyces cerevisiae*. To evaluate this set, which contains 125 genes, it is necessary to consider their 909 annotations. These annotations have 10, 410 representations, which generate 10^216.56 ^combinations. Our approach can evaluate these combinations in only 20 seconds (running on a laptop workstation). Similarly, to evaluate the 125 randomly chosen genes, 10^149.46 ^combinations were generated and evaluated in 29 seconds. Table 1 shows the computational cost of the five largest sets, together with relevant information about the number of annotations, representations and combinations for each set of genes (the first row depicts the sets of genes obtained from metabolic pathways, and the second row shows the sets of randomly chosen genes).

**Table 1:** Computational analysis.

Pathway	Genes	Annotations	Representations	Combinations (*log*_10_)	Time (sec)
sce01100	645	2544	72354	1131.73	1126
		2046	23578	727.49	645

sce01110	235	1005	26745	430.06	114
		716	10502	277.62	108

sce03008	157	312	4090	208.11	3
		557	7883	189.89	41

sce04113	127	884	8850	204.08	19
		450	4917	152.66	33

sce04111	125	909	10410	216.56	20
		461	5801	149.46	29

The five Saccharomyces cerevisiae pathways with the highest number of known genes. For each set of genes (each pathway) the upper row represents real data and the lower row illustrates the pseudorandomly generated data.

The performance of the approach, in terms of the influence of the heuristics on the quality of results, was also analysed. Varying the number of children per node (from 3 to 4) and the number of input genes (from 3 to 10) and randomly generating 100 different trees for each setting, 1600 trees were considered for analysis with and without the heuristics. The use of the heuristic algorithm produced slightly different results in 2.5% of the cases (44 trees), and the average relative error was 0.0005, indicating that the reduction of computational cost does not significantly affect the quality of the results.

## Conclusions

We have introduced a functional dissimilarity measure for gene sets, named GFD (GO-based Functional Dissimilarity) that selects the most cohesive function from a set of input genes. GFD was compared to the most relevant techniques: GS^2 ^and the methods of Wang et al. and Resnik et al. Comparisons were conducted for two different data sets: one based on KEGG pathways and another that was pseudorandomly generated.

To demonstrate the robustness of the method, an ROC analysis was performed for the three GO ontologies to analyse the discriminatory power of the dissimilarity mesures and their sensitivity. In general, GFD is much more accurate for the Molecular Function ontology, and it is equivalent to the Resnik and GS^2 ^methods for the other two ontologies. The area under the ROC curve also shows good performance for both the Biological Process and Molecular Function ontologies (0.99 and 0.98, respectively).

## Authors' contributions

ND conceived the method, designed and implemented the algorithm, and conducted the study. JAR lead the project and participated in writing the manuscript. All authors read, edited and approved the final manuscript.
